# Combining Genomics, Metabolome Analysis, and Biochemical Modelling to Understand Metabolic Networks

**DOI:** 10.1002/cfg.82

**Published:** 2001-06

**Authors:** Oliver Fiehn

**Affiliations:** Max-Planck-Institute of Molecular Plant Physiology 14424 Potsdam Germany

## Abstract

Now that complete genome sequences are available for a variety of organisms, the elucidation of gene functions involved in metabolism necessarily includes a better understanding of cellular responses upon mutations on all levels of gene products, mRNA, proteins, and metabolites. Such progress is essential since the observable properties of organisms – the phenotypes – are produced by the genotype in juxtaposition with the environment. Whereas much has been done to make mRNA and protein profiling possible, considerably less effort has been put into profiling the end products of gene expression, metabolites. To date, analytical approaches have been aimed primarily at the accurate quantification of a number of pre-defined target metabolites, or at producing fingerprints of metabolic changes without individually determining metabolite identities. Neither of these approaches allows the formation of an in-depth understanding of the biochemical behaviour within metabolic networks. Yet, by carefully choosing protocols for sample preparation and analytical techniques, a number of chemically different classes of compounds can be quantified simultaneously to enable such understanding. In this review, the terms describing various metabolite-oriented approaches are given, and the differences among these approaches are outlined. Metabolite target analysis, metabolite profiling, metabolomics, and metabolic fingerprinting are considered. For each approach, a number of examples are given, and potential applications are discussed.


**Comparative and Functional Genomics**
*Comp Funct Genom* 2001; **2**: 155–168.
**DOI:** 10.1002/cfg.82

**Review Article**

### Combining genomics, metabolome analysis, and biochemical modelling to understand metabolic networks

Oliver Fiehn*
*Max-Planck-Institute of Molecular Plant Physiology, 14424 Potsdam, Germany*

*Correspondence to:
O. Fiehn, Max-Planck-Institute of Molecular Plant Physiology, 14424 Potsdam, Germany.
E-mail: fiehn@mpimp-golm.mpg.de

Received: 30 March 2001
Accepted: 5 April 2001

### Abstract

**Now that complete genome sequences are available for a variety of organisms, the elucidation of gene functions involved in metabolism necessarily includes a better understanding of cellular responses upon mutations on all levels of gene products, mRNA, proteins, and metabolites. Such progress is essential since the observable properties of organisms – the phenotypes – are produced by the genotype in juxtaposition with the environment. Whereas much has been done to make mRNA and protein profiling possible, considerably less effort has been put into profiling the end products of gene expression, metabolites. To date, analytical approaches have been aimed primarily at the accurate quantification of a number of pre-defined target metabolites, or at producing fingerprints of metabolic changes without individually determining metabolite identities. Neither of these approaches allows the formation of an in-depth understanding of the biochemical behaviour within metabolic networks. Yet, by carefully choosing protocols for sample preparation and analytical techniques, a number of chemically different classes of compounds can be quantified simultaneously to enable such understanding. In this review, the terms describing various metabolite-oriented approaches are given, and the differences among these approaches are outlined. Metabolite target analysis, metabolite profiling, metabolomics, and metabolic fingerprinting are considered. For each approach, a number of examples are given, and potential applications are discussed. Copyright © 2001 John Wiley & Sons, Ltd.**

**Keywords:** **functional genomics; metabolite profiling; mass spectrometry; metabolism; mathematical modeling**

### Introduction

In all higher organisms, not just plants, the majority of genes have not yet been studied in any experimental depth. Roughly a third of *Arabidopsis thaliana*'s genes have not been assigned putative functions, even based upon sequence similarities with orthologs in other organisms, and only nine percent of all Arabidopsis genes have been studied in any detail. Moreover, many gene assignments are not specific enough to indicate biochemical function, or not detailed enough to define biological roles in a more comprehensive manner [5]. Gene duplications are known to be a major source of rapid evolutionary adaptation, and often result in enzyme isoforms (paralogs) that carry out the same or highly similar functions in different cells or organs within one organism. However, such homologous enzymes (both orthologs and paralogs) may also have quite different substrate specificities or altered kinetic characteristics in order to fulfil new biological roles. This could explain the huge number of up to 200 000 different metabolites estimated to occur in the plant kingdom (D. Strack, personal communication). The full suite of metabolites synthesized by a biological system comprises its metabolome. Such a system can be defined by level of biological organization, such as organism, organ, tissue, cell, or cell compartment levels. In order to determine biological function of a metabolite (and, by association, its cognate enzyme and enzyme-encoding gene), an often-used strategy is to perturb a system

Copyright © 2001 John Wiley & Sons, Ltd.

by systematically introducing genetic alterations and looking for the effect of the perturbation. This can be done by mutating a gene of interest and describing phenotypic effects of this mutation (reverse genetics), or by first identifying an interesting phenotype and then seeking its genetic cause (forward genetics). In both approaches it is essential to describe the phenotype accurately. Several schemes for precisely linking genes to their functions have been suggested, among them metabolic control analysis [15] and the individual analysis of steady-state levels of metabolites [80] in order to comprehensively describe the net result of cellular regulation on the metabolite level. More common, however, are approaches that study cellular responses at the transcript or the protein level (transcriptomics and proteomics, respectively). Current strategies and limitations for the quantitative analysis of cellular responses at all three gene product levels (mRNA, proteins, and metabolites) have been recently summarized in a short review [24], including thoughts on database requirements and informatic tools. Today, transcriptomic approaches seem to give the best coverage of genome level responses. However, due to limitations in analytical precision and high costs, few transcriptomic studies adequately meet rigid statistical requirements. On the other hand, proteomic approaches based on two-dimensional gel electrophoresis are well established in many biological laboratories [78] and are comparatively inexpensive. However, if the full set of proteins separated by 2D gels are to be identified, highly automated systems are needed for cutting spots, digesting proteins, and analysing peptides using mass spectrometry. Therefore, protein identification strategies regularly focus on the most abundant alterations in comparative experiments, such as newly appearing (or completely disappearing) spots, which might lead to erroneous conclusions, since smaller changes in protein abundances can lead to clear alterations in metabolic pathways. Furthermore, low abundance proteins are regularly overlooked [38] as are hydrophobic proteins, which are difficult to resolve using current 2D systems. Quantification of protein abundances can be performed using isotope coded affinity tags with precisions as accurate as 12% relative standard deviations [39], but to date, this technique has not been utilized for proteomic studies that go beyond one-to-one comparative experiments. Compared to transcriptomic and proteomic approaches, analytical techniques for metabolite detection and quantification are far more robust and mature. Analytical precisions may be below 1% relative standard deviations, and dynamic ranges exceed four orders of magnitude. However, *de novo* identification of metabolites is far more difficult than the readout of linear mRNA or protein sequences. Therefore, metabolite analyses have been historically constrained to a number of pre-defined compounds. To describe cellular responses in more depth, several strategies have been developed to answer different questions. These questions are outlined as follows:

(1) In order to study the primary effect of any alteration (e.g. a genetic mutation) directly, an analysis can be restricted exclusively to the substrate and/or the direct product of the corresponding encoded enzyme. In order to improve signal-to-noise ratios, extensive sample cleanup protocols may be used to avoid interferences from major accompanying compounds. This strategy is called *metabolite target analysis* and is mainly used for screening purposes, and for analyses that need extreme sensitivity such as the monitoring of phytohormones.
(2) For investigations of selected biochemical pathways, it is also often not necessary to view the effects of perturbation on all branches of metabolism. Instead, the analytical procedure can be focused on a smaller number of pre-defined metabolites. Sample preparation and data acquisition can be focused on the chemical properties of these compounds with the chance to reduce matrix effects. This process is called *metabolite profiling* (or sometimes *metabolic profiling*). For example, these pre-defined metabolites can be chosen based upon a class of compounds (such as amino acids, organic phosphates, or carbohydrates), or based upon their association with a specific pathway. In the context of drug research or pesticide metabolism, the term *metabolic profiling* is frequently used to describe the metabolic fate of an administered drug.
(3) Due to pleiotropic effects, the effect of a single mutation may lead to the alteration of metabolite levels of seemingly unrelated biochemical pathways. This is especially liable to happen if genes are constitutively overexpressed

Copyright © 2001 John Wiley & Sons, Ltd.

or anti-sense inhibited. A comprehensive and quantitative analysis of all metabolites could help researchers understand such systems. Since such an analysis reveals the metabolome of the biological system under study, this approach should be called *metabolomics*. Both sample preparation and data acquisition must aim at including all classes of compounds, while at the same time assuring high recovery, and experimental robustness and reproducibility. The resolving power of the chosen analytical method must be high enough to maintain sensitivity, selectivity, matrix independence, and universality. Since metabolomic data sets will be complex, adequate tools are needed to handle, store, normalize, and evaluate the acquired data in order to describe the systemic response of the biological system. Furthermore, metabolomic approaches must include strategies to identify unknown metabolites, and analytical tools may even reach out to incorporate models of theoretical biochemical networks.

(4) For functional genomic or plant breeding programmes, as well as for diagnostic usage in industrial or clinical routines, it might not be necessary to determine the levels of all metabolites individually. Instead, a rapid classification of samples according to their origin or their biological relevance might be more adequate in order to maintain a high throughput. This process can be called *metabolic fingerprinting*. Such approaches have occasionally been termed metabonomics, which on the one hand could be mixed up with the completely different goal of metabolomics, and on the other hand with the earlier defined concept of the metabolon, the coordinated channelling of substrates through tightly connected enzyme complexes. Sometimes, metabolic fingerprints have enough resolving power to distinguish between individual signals that can then be related to sample classification. However, it cannot be assumed that such techniques lead to the identification of the most important effects, since major metabolic events might be obscured during data acquisition due to irreproducible matrix effects and lack of analytical resolution and sensitivity.

A number of different metabolomic applications can be imagined. Some are more obvious, such as increasing metabolic fluxes into valuable biochemical pathways by metabolic engineering (e.g. enhancing the nutritional value of foods) or into pathways needed for the production of pharmaceuticals in plants [30]. Other fields of applications are less obvious. For example, metabolomics could be applied in assessments of substantial equivalence of genetically modified organisms [87] when the metabolic phenotypes of well-known cultivars (that are commonly believed to be safe) are compared to transgenic plants. In addition, metabolomic analysis will be of great theoretical value for understanding metabolic responses in more detail. Finally, comprehensive analysis of metabolites could become invaluable in studies that directly aim at detecting biologically active small molecules (such as in drug discovery programmes in which diseased and healthy tissues are compared).

In this review, comparisons are made among the current techniques used to acquire metabolomic data, and strategies to interpret this data to render it useful are discussed.

### Sample preparation

When aiming at the simultaneous detection of the full suite of metabolites in biological samples, the applied methods cannot be restricted to the technical question of which type of data acquisition might be most suitable, but must also seriously consider adequate methods of sample preparation. As a first step, the inherent enzymatic activity of biological samples has to be rapidly stopped by freeze clamping, immediate freezing in liquid nitrogen, or by acidic treatments using perchloric or nitric acid [4]. However, acidic treatments pose severe problems for many analytical methods that follow. Usually, freezing in liquid nitrogen is regarded as the best way to stop enzymatic activity, but if this treatment is used, great care must be taken not to partially thaw tissues before extracting metabolites. This issue can be circumvented using lyophilization (which prevents both enzyme and transporter function), or by immediately adding organic solvents and applying heat, thereby also inhibiting the recovery of enzymatic activity. Using non-aqueous fractionation of lyophilised samples, metabolite levels can be distinguished even from different cellular compartments [29]. Tissues cultures are

Copyright © 2001 John Wiley & Sons, Ltd. 

often directly infused into cold organic solvents, keeping temperatures below –20°C at all times during sample preparation [34]. For plant tissues, sample homogenisation might pose problems. Frozen samples, for example, can be ground using a ball mill in pre-chilled holders [25], or ground directly in an extraction solvent using ultra turrax homogenisers [54]. Other plant organs such as roots, however, prove sometimes to be too hard for ball mills, whereas potato tubers are too soft [63a]. Most frequently, polar organic solvents like alcohols are directly added to homogenized frozen tissues for the extraction of polar components, often followed by non-polar solvents such as dichloromethane for gaining sufficient recovery of lipophilic metabolites.

Any sample preparation protocol must necessarily remain a compromise between complete recovery of some compound classes and avoiding chemical or physical breakdown of more labile metabolites. For example, aromatic compounds might need the input of a reasonable amount of energy into the system (e.g. heat), in order to increase the recovery from (lipophilic) membranes or protein complexes, whereas for other compounds, chemical degradation might occur even at gentle and cold extraction conditions. Furthermore, some compounds (such as polyamines) might need acidic environments for efficient extraction, whereas acidic compounds should best be extracted at slightly basic to neutral conditions. Last, vitamins such as tocopherol are prone to oxidation, and great care must be taken to ensure reproducible extraction of such compounds. Unfortunately, no systematic study has yet been published on metabolomic recoveries and breakdown reactions comparing different techniques of sample preparation, homogenisation, and extraction, although true metabolomic approaches must consider these questions with great care.

### Data acquisition

### Metabolite target analysis

For decades, analytical chemistry has increased the reliability and the sensitivity of detecting predefined compounds in biological tissues. Ultimately, this has lead to the detection of single molecules in single living cells, with great potential of studying biological responses to cellular events *in vivo* [12]. More routine methods have been developed to selectively detect a few members of a compound class while neglecting all others. Polyamines, for example, are believed to be involved in a number of processes important for plant systems, such as drought stress, and various analytical methods are available for their reliable quantification in plant material [10]. Vitamins remain the objects of ongoing analytical research [6], especially when the simultaneous analysis of different isoforms is required [71]. On another note, long-studied compounds might still hold some challenges, such as the reliable detection of metabolites that appear simultaneously in the oxidized and reduced form, such as glutathion [52], or in different stereoisomers, such as zeaxanthins [17]. Most demanding remains the analysis of trace compounds in extremely complex matrices, such as phytohormones in plants. A variety of protocols have been developed for the detection of indole-3-acetic acid [60], for abscisic acid [19], and for indole-3-pyruvate [74], and methods for phytohormone analysis will almost certainly be further improved in coming years to achieve better detection limits and easier sample clean-ups.

Target analysis will remain the most wide-spread technique, with applications in all areas of biological research. However, for comparative analysis in functional genomics studies, target analysis is only of limited use, since the levels of the target analytes might be altered by unexpected effects that can not be understood without more comprehensive approaches. Therefore, a broader analysis of metabolic alterations is needed to limit over-interpretation of data. In the following section, the concepts and results of multi-target profiling approaches and non-biased data acquisition will be reviewed.

### Metabolite profiling

Since the late 1960's, improved chromatographic methods have made peak identifications possible relying solely on chromatography. When coupled to sensitive detectors, these analytical methods were soon applied to urine samples and plant tissues to profile important compound classes such as amino acids [2]. By including compounds with known retention times, shifts in absolute retention times could be taken into account. In one application of this method, up to 155 organic acids were detected in order to diagnose human diseases in a clinical routine [75,76]. Mass spectrometry offered an

Copyright © 2001 John Wiley & Sons, Ltd. 

additional and completely independent method for compound identification. By coupling gas chromatography to mass spectrometry (GC/MS), fifty different human diseases could be diagnosed simultaneously [41]. Today, computational constraints facilitate a more automated and more reliable categorization of human metabolic disorders [49] and cancer-related tissues [48].

For automated metabolite identification, reliable information on both retention time and mass spectra is required. However, mass spectra of metabolites can be dominated by co-eluting compounds in complex chromatograms, and may be obscured at trace levels by chemical noise. To allow high threshold values for mass spectral quality in routine identifications, mass spectra therefore need to be purified. By using mass spectral deconvolution software, peak identification was possible for 68 target compounds for the rapid detection of inborn errors [40] when comparing samples from diseased and healthy children.

Less work has been done on the comparative analysis of profiling plant compounds. The simultaneous determination of carbohydrates, sugar alcohols, acids, sterols, and amino acids by GC/MS was first explored by Sauter *et al.* [64] for comparing the effects of pesticide applications on plants. Due to the lack of sample pre-fractionation, the chromatograms were heavily crowded, and less abundant metabolites such as lysine were easily missed. By restricting the analysis to polar compounds, derivatisation protocols were further optimised [1], and profiles of polar metabolites in apricots were generated [45]. More systematically, Roessner *et al.* [63a,b] evaluated the utility of GC/MS measurements for the analysis of polar metabolites in potato tubers. However, in different organisms (and also, in different organs of the same organism), biochemical pathways may be quite differently organized, and pathways could not be as conserved as textbooks suggest. Therefore, the actual biochemical pathways must be reinvestigated using modern analytical tools. For example, GC/MS can be used to investigate metabolic networks consisting of a small number of metabolites using stable isotopes and profiling the fractional enrichment [14].

For some compound classes, such as bis- and trisphosphates or lipids, liquid chromatography (LC) is the method of choice for separation. By measuring the absorption of ultraviolet light (UV), profiles of aromatic and de-saturated organics can be acquired, such as carotenes, xanthophylls, ubiquinones, tocopherols, and plastoquinones. LC/UV has successfully been used to characterize transgenic and mutant tomato genotypes and for screening *Arabiodpsis* mutants [27]. However, compared to UV detection, mass spectrometers are clearly more versatile and are capable of not only analysing isoprenoids and aromatics, but also compounds without UV absorbing moieties (such as oligosaccharides). Since the beginning of the 1990's, electrospray ionisation has offered a robust and versatile interface to connect liquid chromatography and mass spectrometry. For compound classes such as sugar polyols, it has been shown that its analytical precision is high enough for reliable quantifications, if stable isotope labelled compounds are used as internal references [69]. For other classes of compounds such as ceramides, LC/MS showed detection limits in the femtomolar range for analysis of cultured T-cells [37,59]. The highest absolute sensitivity for metabolite profiling can be gained by connecting capillary electrophoresis to laser-induced fluorescence detection. With this technique, steroids could be quantified in the attomolar range, compared to femtomolar sensitivities when coupled to mass spectrometry.

Most frequently, the term metabolic profiling refers to the catabolic degradation of a certain compound in an organism. In order to study such degradation pathways comprehensively, several analytical approaches may be followed in parallel. Beuerle and Schwab [8] investigated the degradation of linoleic acid in stored apples using GC/MS, LC/MS/MS and LC in conjunction with radioactivity detection. Even more frequently, metabolic profiles are determined in pharmaceutical research in order to follow the metabolic fate of administered drugs. A typical example of this is the elucidation of the biochemical pathways of propanolol degradation in rats using LC/MS/MS [7]. This can be coupled to bioassay directed fractionation, such as the binding affinity of catabolites to specific receptors [51], in order to gain information about the biological (or toxicological) relevance of catabolites.

### Metabolomics

The obvious next step in metabolic network analysis is to try to determine metabolic snapshots in a broad and comprehensive way. In metabolomic approaches, any bias against a certain class of

Copyright © 2001 John Wiley & Sons, Ltd. 

compounds must be avoided. Instead, biological importance is defined by evaluating relative changes of metabolite levels in comparative experiments. It is of utmost importance, therefore, that the abundance of any metabolite can be directly compared from one sample to the next, which makes the use of stable isotope standards to cope with potential matrix effects highly advantageous. Furthermore, it is probably wise to use fractionation steps (like lipophilic/hydrophilic separations) and chromatographic separations in order to minimize the number of compounds that reach the analytical device simultaneously. To demonstrate the power of such an approach, a profile with over 150 detectable peaks in the base peak chromatogram is shown for the polar phase of potato leaves (Figure 1). In a proof-of-concept study, such GC/MS analysis was chosen by Fiehn *et al.* [26] to characterize plant mutants using a two-phase fractionation protocol. 326 polar and lipophilic compounds were analysed, half of which had no assigned chemical structure. Two mutants were compared to their parental genotypic backgrounds, and metabolic phenotypes were assigned by clustering the acquired data according to the sample origin. However, analysis was restricted to abundant peaks, and, almost certainly, a number of trace compounds will have been overlooked by this approach. GC/MS analyses were also used for studying metabolic phenotypes in wild type and transgenic potato tubers, using 86 abundant peaks selected from the chromatograms, followed by clustering the data according to metabolic phenotypes [63b]. In this paper, however, quantitative alterations of only a few unidentified metabolites were taken into account, and presumably, an even higher number of peaks remained undetermined. Another approach to identifying gene functions using extended chromatographic analysis was performed by Tweedale *et al.* [81]. After growing wild type and mutant *E. coli* strains in minimal media and $^{14}C$-labelled glucose, the 70 most abundant metabolites were separated on two-dimensional thin layer chromatography. Relative quantification of metabolites by radioactivity

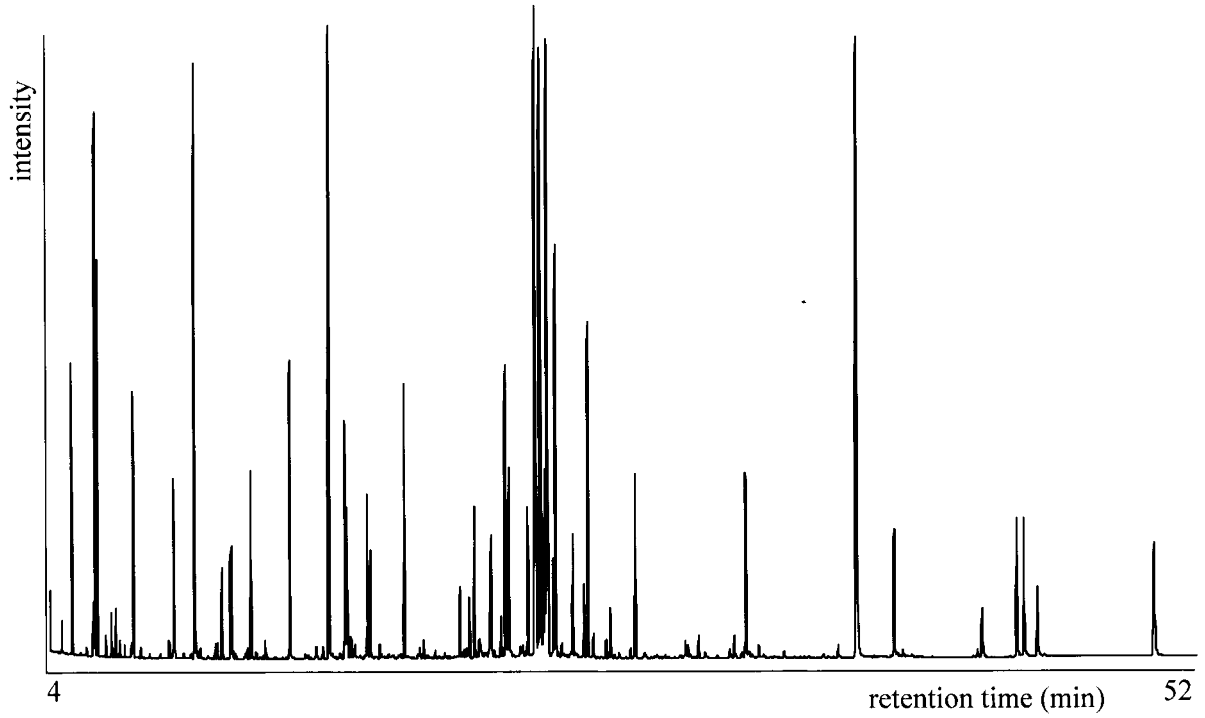

**Figure 1.** Polar phase of Solanum tuberosum leaves, analysed by GC/quadrupole MS (unpublished results). Inspection of peaks apparent in the base peak chromatogram results in some 150 distinct metabolites. Abundant peaks in the middle of the chromatogram are monosaccharides, followed by disaccharides (sucrose being the largest), and raffinose at the end of the chromatogram. Trimethylsilylated hydroxy- and amino acids are eluted in the first third of the profile

Copyright © 2001 John Wiley & Sons, Ltd. 

detection showed reproducible alterations in metabolite pools (among them from unidentified metabolites), depending on culture conditions. However, changes in metabolite pools could only partly be ascribed to known control functions of the mutated gene.

In metabolomic analysis of comparative experiments, major changes in metabolite levels will almost certainly include unidentified peaks. Therefore, metabolomic research should include approaches aimed at elucidating chemical structures, for example by combining liquid chromatography with nuclear magnetic resonance detection (NMR) and mass spectrometry [86]. For GC separations, however, *de novo* identification strategies are less straightforward. For polar components, for example, chemical derivatisation that hampers structural investigation is needed, especially if hard ionisation techniques such as electron impact ionization are applied. In order to gain information about the intact molecule, derivatisation agents can be used that result in characteristic pseudo molecular ions. Using this approach, 30 uncommon plant metabolites were identified after calculation of elemental compositions and database queries [25]. However, compounds larger than monosaccharides could not be detected using this method due to decreased volatility of the corresponding derivatives and incomplete derivatisation due to steric hindrance of the reagent. In conclusion, metabolomic approaches based on GC/MS need better procedures for identifying unknown peaks. This could potentially be achieved by softer ionisation techniques (such as chemical ionisation), and by combining information derived from mass spectral fragmentation patterns, isotope ratios, exact masses, structure generators, and (bio)chemical databases.

As pointed out above, high throughput analyses for functional genomics also need an automatic procedure to assign an indicator of the reliability of a compound match. This was achieved by developing an automatic mass spectral deconvolution and identification software (AMDIS) by Stein [72]. This software is capable of computing purified mass spectra from the elution profile of a compound by deconvolution of the overlapping mass spectra of its neighbouring compounds (or, background ions that stem from chemical noise). Using the deconvoluted mass spectra, peak identities are confirmed by searching mass spectral libraries. Halket *et al.* [40] used this software to enhance the reliability of peak identifications in GC/MS runs, but did not take the total number of peaks into account.

Today, analytical methods such as GC/MS, NMR, and LC/UV/MS are reliable and robust enough to be used as workhorses in biological laboratories, yet sample preparation protocols seem to contain the most error prone steps (that ultimately might cause irreproducible or artefactual results). In metabolomic approaches, all protocols have intrinsic biases for and against chemically different classes of metabolites. Therefore, recoveries and reproducibilities cannot be as high as in metabolite profiling or metabolite target analyses. Instead, metabolomic analyses have to be regarded as 'quick-and-dirty' methods, that aim to be as comprehensive and as fast as possible, but that cannot insure the precise quantification of each and every metabolite.

### Metabolic fingerprinting

Comprehensive metabolomic analyses cannot be achieved without pre-fractionation steps, chromatographic separation, and use of different analytical instruments. Therefore, each sample has to be portioned into a (limited) number of aliquots, reducing the total sample throughput. If a higher number of samples need to be analysed, for example for rapid classifications, even faster methods can be applied that completely refrain from sample clean-up steps or time consuming chromatography. This might be needed for diagnostic purposes in the clinical routine, for product quality controls, or for analysing large mutant collections in functional genomics programmes. The bottom line of metabolic fingerprinting is to obtain enough information to unravel (otherwise hidden) metabolic alterations, without aiming to get quantitative data for all biochemical pathways. Therefore, the resolution of the analytical devices must be high enough to handle critical information. Such devices as nuclear magnetic resonance, mass spectrometry, or Fourier transform infrared spectroscopy (FT-IR) provide this resolution. Using a combination of pyrolysis mass spectrometry and FT-IR, bacterial species have been classified using novel programming tools, resulting in potential biomarkers then used to rapidly distinguish among these species [35]. A similar approach was taken by Smedsgaard and Frisvad [70], who used direct infusion of crude fungal extracts into MS/MS instruments in order to

Copyright © 2001 John Wiley & Sons, Ltd.

classify ten different fungal species. NMR was used to detect effects of toxins on rats via the direct analysis of dried urine samples, and principle components analysis for classification of metabolic alterations [62]. However, metabolic fingerprinting can easily be over-interpreted, since signals suitable for distinguishing among samples might not be biologically relevant, or might not be applicable when distinguishing among samples from other species (or situations). For example, Warne *et al.* [84] studied metabolic effects by NMR after dosing earthworms with toxins. By pattern recognition, they noted elevated levels of glucose, citrate, and succinate as potential biomarkers for toxicity. However, there are clearly a lot of situations where intermediates of the TCA cycle become elevated, and generalisations about the suitability of this method for detecting toxic effects should be avoided. For example, differences in the levels of TCA intermediates were also found by NMR analyses when investigating urine samples from mutant mice [28]. In the realm of functional genomics, NMR was used to detect metabolic phenotypes in yeast mutants that did not show obvious visible phenotypes. However, the informative power of NMR was not sufficient in this instance to quantify individual metabolite levels; enzymatic analysis had to be applied additionally [58].

Apart from NMR and MS, infrared spectroscopy has also been used to find differences in comparative experiments. For example, tomato fruits from plants grown under salinity stress can be distinguished from those grown under normal conditions based on so-called genetic programming [43]. Obviously, all approaches to metabolic fingerprinting have made use of sophisticated informatic tools in order to deconvolute raw analytical data. However, Gilbert *et al.* [31] emphasized that only genetic programming gives interpretable equations for the underlying reasons leading to final classification results.

NMR, low-resolution MS, and FT-IR all lack resolving power to distinguish all the metabolites in a single spectrum. To date, no study has been published that utilizes the enormous resolving power of Fourier-transform ion cyclotron resonance mass spectrometry (FT-MS). Theoretically, all small metabolites of an organism could be analysed simultaneously using this approach (without any chromatography), since the FT-MS resolution of $R > 100\,000$ allows the unambiguous detection of metabolites that are only 0.005 Da apart, and the accurate masses of these metabolites could be used for *de novo* identification. However, such an approach would face some severe limitations. First, isomers having identical elemental compositions (such as fructose and glucose) could not be distinguished. Second, matrix effects could cause severe alterations in electrospray ionisation efficiency by ion suppression. And finally, ion repulsion in the cyclotron cell could occur, which would clearly hamper high resolution and accurate mass analyses. Nevertheless, FT-MS seems prone to be used for metabolic fingerprinting, and it might be a powerful tool for rapidly detecting major metabolic differences when screening mutant collections.

### Data interpretation

### Pattern recognition

Regardless of which analytical method is used, metabolomic analyses, as well as profiling major events by fingerprinting, will result in large collections of raw data. As long as more than subtle metabolic changes are expected, the analysis of metabolic profiles should definitely result in clear clusters according to the design of the comparative experiment, i.e. mutant/wild type, healthy/diseased, young/old, etc. If such comparisons cannot be verified by clustering tools, the data might be too noisy to be further analysed. The lack of inherent information might either be trivial (important alterations in metabolite levels could be missed), or errors might be introduced during sample preparation steps or by data acquisition itself. Next, any subgroups within the major clusters must be tested to insure that classification occurred as per the intended experimental set-up. Again, such subclusters might be generated either by systematic errors in sample preparation or data acquisition, or by random errors such as slight differences among culture treatments, even if the investigator believed treatments to be under control. Both reasons cannot be fully excluded in metabolomic analyses of comparative biological experiments, since there are simply too many factors that could cause subtle changes in clustering results. In Figure 2, a potential result of a hierarchical clustering analysis is demonstrated for a hypothetical experiment. Samples are easily classified according their origin, A or B,

Copyright © 2001 John Wiley & Sons, Ltd. 

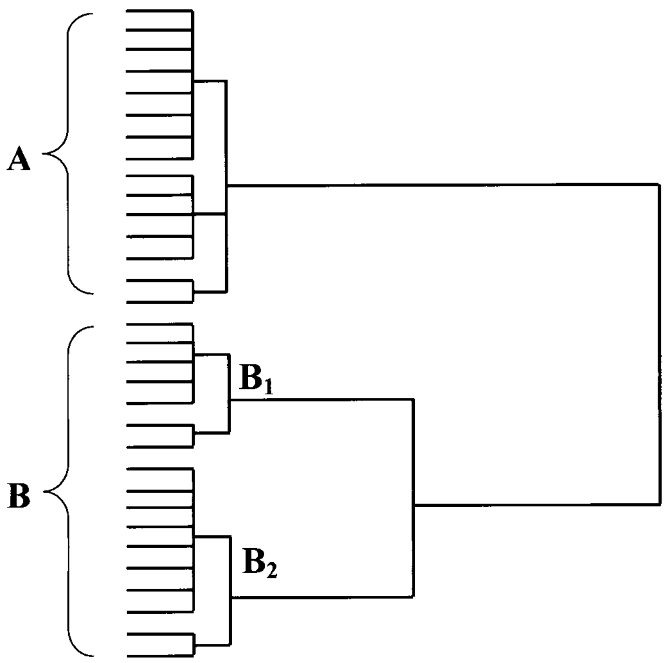

**Figure 2.** Cluster analysis of a hypothetical experiment. Hierarchical clustering of the samples using Euclidean distances for all metabolites might result in the expected separation of samples from origin A (such as wild type samples) and from origin B (such as mutant samples). In this example, B samples fall into two sub-groups. $B_1$ and $B_2$, as indicated by the length of the lines however, two sub-clusters can be seen within the B population. Once such a subgroup is found within B, it is not statistically sound to treat B as one population and to compare it to A, by Student's *t* test for example. Numerous approaches exist for statistical analysis, such as multiple analysis of variance (MANOVA) or analysis of frequency distributions, but great care should be taken to meet the statistical requirements for such tests. Whenever possible, experienced statisticians should be asked to evaluate the best experimental design in order to answer a specific question. More important than clustering metabolic phenotypes or calculating alterations in average metabolite levels might be indications of further relationships within metabolomic data sets. Yet, there is not much experience in analysing such hidden relationships. The current paradigm is that cluster analysis of linear relationships of variables (e.g. gene expression) might lead to candidate genes with similar biological roles in cellular processes [22]. Bittner *et al.* [9] briefly summarized current approaches to analyse relationships in mRNA expression data sets, and investigators using metabolomic data analysis might learn from these experiences. The authors conclude that considerable efforts have been made to cluster linear one-to-one correlations, but the investigation of non-linear responses may be much more biologically important. Non-linear response curves could be investigated using more sophisticated information tools, such as the concept of mutual information [68]. Furthermore, non-trivial results may also be obtained by applying other concepts such as rule-based learning methods. Gilbert *et al.* [32] have utilized a variant of such supervised learning algorithms, genomic computing, to build new biological hypotheses from the re-analysis of mRNA expression data deposited in publicly accessible data banks. Very likely, the best we can get from bioinformatic analyses of large-scale data sets is the generation of new hypotheses, and information concerning how much evidence was found supporting each of the hypotheses. Such information can then be the starting point of hypothesis generation. Other groups can then work to falsify or substantiate hypotheses using classical biochemistry and molecular biology.

### Metabolic networks

To further test the biological relevance of hypotheses gained from metabolomic data sets, these

Copyright © 2001 John Wiley & Sons, Ltd.

data should be compared to predictions made either by searching connections to known biochemical pathways, or by using prediction models based on mathematical calculations from biochemical kinetics or stoichiometries. For the former, classical textbooks certainly give a good start. However, as large networks are generated, a broader view on metabolic interactions will be needed. Within the publicly available genomic data base KEGG [46,53], links to encoded enzymatic pathways can be found with maps visualizing standard metabolic pathways of different organisms. However, KEGG has only a partial overlap with other enzymatic or metabolic databases and it is worth looking into BRENDA [11], WIT [85], and PathDB [56].

Another possibility when comparing metabolic networks is to follow theoretical considerations. Two basic approaches can be found in literature: first, metabolic fluxes can be calculated from experimental knowledge of enzyme kinetics in a method called metabolic control analysis [4,44,77]. Secondly, metabolic pathways can be calculated to be feasible or not by considering the stoichiometry of enzymatic reactions [36,57]. Below, both approaches are briefly evaluated for their applicability to metabolomic research.

Metabolic control analysis has regularly been applied to forward the aim of increasing carbon flux through certain biochemical pathways in biotechnological applications [13,33]. These authors emphasized that relative directions and relative intensities of metabolite fluxes must be determined in order to understand even small metabolic networks in full. One way to measure such fluxes is by adding isotopically labelled compounds (often by growing cultures on $^{13}$C-labelled Glucose) and following the kinetics of isotope distribution by means of NMR or MS [73,82].

By analysing of the fine structure of NMR spectra, the positions of incorporated $^{13}$C atoms can be determined, enabling the mathematical modelling of the contribution of different pathways to the metabolic cycles [50,55,65]. In plant systems, however, the situation is even more complicated. Plant metabolism is heavily split among several cellular compartments, and a range of methods must be combined to fully elucidate metabolic fluxes into certain pathways [61]. Using LC/fluorescence, off-line radioactivity measurements, and NMR, such metabolite fluxes were successfully elucidated in maize root tips after application of $^{13}$C- and $^{14}$C-labelled glucose [18]. For selected organs like potato tubers, Thomas *et al.* [79] were able to successfully explain enzymatic influence in certain pathways using metabolic control analysis. Further limitations of metabolic control analysis were reviewed by Kell and Mendes [47] who emphasized that biochemical predictions using metabolic control analysis is now only achievable for small, comparatively simple pathways, and that it can only be applied if no drastic changes in enzymatic activities occur. Therefore, metabolic control analysis does not seem to be directly applicable to metabolomic data sets in comparative experiments, which are often designed to study dramatic alterations like mutant/wild type comparisons. Alternatively, metabolic fluxes may also be modeled from *in vitro* determined enzyme kinetics. However, the *in vivo* kinetics of these enzymes might be quite different. Additionally, the bottom line of metabolic control is independent enzyme action. However, it is unclear if this assumption holds true for all cellular processes or if the coordinated action of multiple enzymes may in fact be a more realistic maxim [83].

In the second approach towards prediction of metabolic networks, the enzymatic reactions are further simplified by taking only the stoichiometries of substrates and products into account, in order to calculate feasible and optimal metabolic flux directions. The only constraints that are used for such calculations (also called flux balance analyses) are systemic mass balances and reaction capacities, while neglecting constraints given by mRNA or protein expression, or enzyme kinetics.

Such models can be computed from functional assignments of genes for any organism, and no further experimental data are needed. Therefore, large metabolic networks can be built from matrix correlations of overall substrate-product stoichiometry, but almost certainly, the models derived from such calculations will lack prediction power due to the lack of additional experimental evidence concerning cellular compartmentalisation and *in vivo* kinetics. Nevertheless, non-obvious links in biochemical pathways can be found by pure computer simulations [16]. This is especially true if each pathway is reduced to a set of strongly co-operating enzymes, as developed in the concept of 'elementary flux modes' by Schuster *et al.* [66,67]. In this approach, biochemical pathways are not defined by the interpretation of individual scientists, but are

Copyright © 2001 John Wiley & Sons, Ltd.

purely based on computing the thermodynamic and stoichiometric feasibility of enzymatic conversions between arbitrarily chosen sets of metabolites. In two break-through papers, growth rate data of *E.coli* mutants were compared to predictions made from stoichiometric matrices. In 86% of the studied cases, the effects of gene knockouts in *E.coli* could be correctly predicted when compared to data given in literature [20,21].

Apart from stoichiometric approaches, standard biochemical pathways can be considered with even further simplifications. Each possible substrate-product conversion may be regarded as an edge in visualizations of metabolic networks. Fell and Wagner [23] have suggested that metabolic networks generated by such simplifications are scale-free networks. Therefore, they could potentially be used to analyse the inherent connections, for example in evolutionary studies. This approach has also been followed in two studies from the Barabási group [3,42], in which the authors showed that metabolism is generally organized in such scale-free networks, which might be less prone to malfunctions caused by errors like random mutations.

### Conclusions

Metabolomic analyses have only just begun, but it is clear that the analytical challenges associated with the relative quantification of metabolites can be met more easily than those associated with the *de novo* identification of unknown metabolites. However, a combination of results from in-depth characterization of genetically altered organisms using transcriptomics, proteomics, metabolomics, and accurate descriptions of developmental phenotypes is now more feasible than was imagined just five years ago. Chasing the dream of comprehensively understanding living organisms will also require improved data mining tools, and better tools for integrating the results of experimentally determined molecular phenotypes with predictions made by computational simulations of cellular networks. For example, right now it is difficult to track the primary effects of mutations using metabolic analyses. However, theoretically it should be possible to link observed changes in metabolic pathways to the underlying genetic alterations via the enzymes involved in these pathways. So far, however, no results have been published on how to generate hypotheses about novel gene functions by metabolite analysis. Eventually, small biological laboratories will be unable to combine all the genetic, analytical, and computational resources in their institutions. Therefore, larger institutions should face the responsibilities of building up analytical resource centres and of creating publicly accessible metabolomic databases similar to genomic sequence repositories.

### Acknowledgement

I would like to thank Megan McKenzie for editing the manuscript.

### References

1. Adams MA, Chen ZL, Landman P, Colmer D. 1999. Simultaneous determination by capillary gas chromatography of organic acids, sugars, and sugar alcohols in plant tissue extracts as their trimethylsilyl derivatives. *Anal Biochem* **266**: 77–84.
2. Adams RF. 1974. Determination of amino acid profiles in biological samples by gas chromatography. *J Chromatogr* **95**: 189–212.
3. Albert R, Jeong H, Barabási AL. 2000. Error attack tolerance of complex networks. *Nature* **406**: 378–381.
4. ap Rees T, Hill SA. 1994. Metabolic control analysis of plant metabolism. *Plant Cell Env* **17**: 587–599.
5. Attwood TK. 2000. The babel of bioinformatics. *Science* **290**: 471–473.
6. Bates CJ. 1997. Vitamin analysis. *Ann Clin Biochem* **34**: 599–626.
7. Beaudry F, Le Blanc JCY, Coutu M, Ramier I, Moreau JP, Brown NK. 1999. Metabolite profiling study of propranolol in rat using LC/MS/MS analysis. *Biomed Chromatogr* **13**: 363–369.
8. Beuerle T, Schwab W. 1999. Metabolic profile of linoleic acid stored in apples: formation of 13(R)-hydroxy-9(Z),11(E)-octadecadienoic acid. *Lipids* **34**: 375–380.
9. Bittner M, Meltzer P, Trent J. 1999. Data analysis and integration: of steps and arrows. *Nat Genet* **22**: 213–215.
10. Bouchereau A, Guenot P, Lather F. 2000. Analysis of amines in plant materials. *J Chromatogr* **B 747**: 49–67.
11. BRENDA **http://www.brenda.uni-koeln.de/**
12. Byassee TA, Chan WCW, Nie SM. 2000. Probing single molecules in single living cells. *Anal Chem* **72**: 5606–5611.
13. Christensen B, Nielsen J. Metabolic network analysis: a powerful tool in metabolic engineering. In *Advances in Biochemical Engineering and Biotechnology*, Scheper T (ed). Springer: Berlin; 210–231.
14. Christensen B, Nielsen J. 2000. Metabolic network analysis of Penicillium chysogenum using $^{13}$C-labelled glucose. *Biotechnol Bioeng* **68**: 652–659.
15. Cornish-Bowden A, Cárdenas ML. 2000. From genome to cellular phenotype – a role for metabolic flux analysis? *Nat Biotechnol* **18**: 267–268.

Copyright © 2001 John Wiley & Sons, Ltd. 

16. Cornish-Bowden A, Eisenthal R. Computer simulations as a tool for studying metabolism and drug design. In *Technological and medical implications of metabolic control analysis*, Cornish-Bowden AJ, Cárdenas ML (eds). Kluwer Academic Publishers: Dordrecht; 165–172.
17. Dachtler M, Glaser T, Kohler K, Albert K. 2001. Combined HPLC-MS and HPLC-NMR on-line coupling for the separation and determination of lutein and zeaxanthin stereoisomers in spinach and in retina. *Anal Chem* **73**: 667–674.
18. Dieuaide-Noubhani M, Raffard G, Canioni P, Pradett A, Raymond P. 1995. Quantification of compartmented metabolic fluxes in maize root tips using isotope distribution from $^{13}C$- or $^{14}C$-labeled glucose. *J Biol Chem* **22**: 13147–13159.
19. Duffield PH, Netting AG. 2001. Methods for the quantitation of abscisic acid and its precursors from plant tissues. *Anal Biochem* **289**: 251–259.
20. Edwards JS, Ibarra RU, Palsson BO. 2001. *In silico* predictions of *Escherichia coli* metabolic capabilities are consistent with experimental data. *Nat Biotechnol* **19**: 125–130.
21. Edwards JS, Palsson BO. 2000. The *Escherichia coli* MG 1655 in silico metabolic genotype: its definition, characteristics, and capabilities. *Proc Natl Acad Sci U S A* **97**: 5528–5533.
22. Eisen MB, Spellmann PT, Brown PO, Botstein D. 1998. Cluster analysis and display of genome-wide expression patterns. *Proc Natl Aca Sc U S A* **95**: 14863–14868.
23. Fell DA, Wagner A. 2000. The small world of metabolism. *Nat Biotechnol* **18**: 1121–1122.
24. Fiehn O, Kloska K, Altmann T. 2001. Integrated studies on plant biology using multiparallel techniques. *Curr Opin Biotechnol* **12**: 82–86.
25. Fiehn O, Kopka J, Trethewey RN, Willmitzer L. 2000a. Identification of uncommon plant metabolites based on calculation of elemental compositions using gas chromatography and quadrupole mass spectrometry. *Anal Chem* **72**: 3573–3580.
26. Fiehn O, Kopka J, Dörmann P, Altmann T, Trethewey RN, Willmitzer L. 2000b. Metabolite profiling represents a novel and powerful approach for plant functional genomics. *Nat Biotechnol* **8**: 1157–1161.
27. Fraser PD, Pinto MES, Holloway DE, Bramley PM. 2000. Application of high-performance liquid chromatography with photodiode array detection to the metabolic profiling of plant isoprenoids. *Plant J* **24**: 551–558.
28. Gavaghan CL, Holmes E, Lenz E, Wilson ID, Nicholson JK. 2000. An NMR-based metabonomic approach to investigate the biochemical consequences of genetic strain differences: application to the C57BL10J and Alpk : ApfCD mouse. *FEBS Lett* **484**: 169–174.
29. Gerhardt R, Heldt HW. 1984. Measurement of subcellular metabolite levels in leaves by fractionation of freeze-stopped material in nonaqueous media. *Plant Physiol* **75**: 542–547.
30. Giddings G, Allison G, Brooks D, Carter A. 2000. Transgenic plants as factories for biopharmaceuticals. *Nat Biotechnol* **18**: 1151–1155.
31. Gilbert RJ, Goodacre R, Woodward AM, Kell DB. 1997. Genetic programming: a novel method for the quantitative analysis of pyrolysis mass spectral data. *Anal Chem* **69**: 4381–4389.
32. Gilbert RJ, Rowland JJ, Kell DB. Genomic computing: explanatory modelling for functional genomics. In *Proceedings of the Genetic and Evolutionary Computation Conference*. Whitley D, Goldberg D, Cantú-Paz E (eds). Morgan Kaufman: San Francisco; 551–557.
33. Gombert AK, Nielsen J. 1999. Mathematical modelling of metabolism. *Curr Opin Biotechnol* **1**: 180–186.
34. Gonzalez B, Francois J, Renaud M. 1997. A rapid and reliable method for metabolite extraction in yeast using boiling buffered ethanol. *Yeast* **13**: 1347–1356.
35. Goodacre R, Shann B, Gilbert RJ, *et al*. 2000. Detection of the dipicolinic acid biomarker in *Bacillus* spores using curie-point pyrolysis mass spectrometry and fourier transform infrared spectroscopy. *Anal Chem* **72**: 119–127.
36. Goryanin I, Hodgman TC, Selkov E. 1999. Mathematical simulation and analysis of cellular metabolism and regulation. *Bioinformatics* **15**: 749–758.
37. Gu M, Kerwin JL, Watts JD, Aebersold R. 1997. Ceramide profiling of complex lipid mixtures by electrospray ionisation mass spectrometry. *Anal Biochem* **24**: 347–356.
38. Gygi SP, Corthals GL, Zhang Y, Rochon Y, Aebersold R. 2000. Evaluation of two-dimensional gel electrophoresis-based proteome analysis technology. *Proc Natl Acad Sci U S A* **97**: 9390–9395.
39. Gygi SP, Rist B, Gerber SA, Turecek F, Gelb MH, Aebersold R. 1999. Quantitative analysis of complex protein mixtures using isotope-coded affinity tags. *Nat Biotechnol* **17**: 1112–1118.
40. Halket JM, Przyborowska A, Stein SE, Mallard WG, Down S, Chalmers RA. 1999. Deconvolution gas chromatography mass spectrometry of urinary organic acids – Potential for pattern recognition and automated identification of metabolic disorders. *Rapid Commu Mass Spectrom* **13**: 279–284.
41. Jellum E, Kvittingen EA, Stokke O. 1988. Mass spectrometry in diagnosis of metabolic disorders. *Biomed Enviro Mass Spectrom* **16**: 57–62.
42. Jeong H, Tombor B, Albert R, Oltvai ZN, Barabási AL. 2000. The large-scale organization of metabolic networks. *Nature* **406**: 651–654.
43. Johnson HE, Gilbert RJ, Winson MK, *et al*. 2000. Explanatory analysis of the metabolome using genetic programming of simple, interpretable rules. *Genet Program Evolv Mach* **1**: 243–258.
44. Kacser H, Burns JA. 1973. The control of flux. *Symp Soc Exp Biol* **27**: 65–105.
45. Katona ZF, Sass P, Molnár-Perl I. 1999. Simultaneous determination of sugars, sugar alcohols, acids and amino acids in apricots by gas chromatography-mass spectrometry. *J Chromatogr A* **847**: 91–102.
46. KEGG **http://www.genome.ad.jp/kegg/**
47. Kell DB, Mendes P. Snapshots of systems. In *Technological and Medical Implications of Metabolic Control Analysis*, Cornish-Bowden AJ, Cárdenas ML (eds). Kluwer Academic Publishers: Dordrecht; 3–25.
48. Kim K-R, Park H-G, Paik M-J, *et al*. 1998. Gas chromatographic profiling of urinary organic acids from uterine myoma patients and cervical cancer patients. *J Chromatogr B* **712**: 11–22.
49. Kimura H, Yamamoto T, Seiji Y. 1999. Automated metabolic profiling and interpretation of GC/MS data for

Copyright © 2001 John Wiley & Sons, Ltd. 

organic academia screening: a personal computer-based system. *Tohuku J Exp Med* **18**: 317–344.

50. Klapa MI, Park SM, Sinskey AJ, Stephanopoulos G. 1999. Metabolite and isotopomer balancing in the analysis of metabolic cycles: I. *Theory Biotechnol Bioeng* **62**: 375–391.
51. Lim HK, Stellingweif S, Sisenwine S, Chan KW. 1999. Rapid drug metabolite profiling using fast liquid chromatography, automated multiple-stage mass spectrometry and receptor-binding. *J Chromatogr A* **831**: 227–241.
52. Manna L, Valvo L, Betto P. 1999. Determination of oxidized and reduced glutathione in pharmaceuticals by reversed-phase high-performance liquid chromatography with dual electrochemical detection. *J Chromatogr A* **846**: 59–64.
53. Ogata H, Goto S, Sato K, Fujibuchi W, Bono H, Kanehisa M. 1999. KEGG: Kyoto encyclopedia of genes and genomes. *Nucleic Acids Res* **27**: 29–34.
54. Orth HCJ, Rentel C, Schmidt PC. 1999. Isolation, purity analysis and stability of hyperforin as a standard material from Hypericum perforatum L. *J Pharm Pharmcol* **51**: 193–200.
55. Park SM, Klapa MI, Sinskey AJ, Stephanopoulos G. 1999. Metabolite and isotopomer balancing in the analysis of metabolic cycles: II. Applications. *Biotechnol Bioeng* **62**: 392–401.
56. PathDB **http://www.ncgr.org/software/pathdb/**
57. Pfeiffer T, Sánchez-Valdenebro I, Nuno JC, Montero F, Schuster S. 1999. Metatool: for studying metabolic networks. *Bioinformatics* **15**: 251–257.
58. Raamsdonk LM, Teusink B, Broadhurst D, *et al.* 2001. A functional genomics strategy that uses metabolome data to reveal the phenotype of silent mutations. *Nat Biotechnol* **19**: 45–50.
59. Raith K, Zellmer S, Lasch J, Neubert RHH. 2000. Profiling of human stratum corneum ceramides by liquid chromatography-electrospray mass spectrometry. *Anal Chim Acta* **418**: 167–173.
60. Ribnicky DM, Cooke TJ, Cohen JD. 1998. A microtechnique for the analysis of free and conjugated indole-3-acetic acid in milligram amounts of plant tissue using a benchtop gas chromatograph mass spectrometer. *Planta* **204**: 1–7.
61. Roberts JKM. 2000. NMR adventures in the metabolic labyrinth within plants. *Trends Plant Sci* **5**: 30–34.
62. Robertson DG, Reily MD, Sigler RE, Wells DF, Paterson DA, Braden TK. 2000. Metabonomics: Evaluation of nuclear magnetic resonance (NMR) and pattern recognition technology for rapid in vivo screening of liver and kidney toxicants. *Toxicol Sci* **57**: 326–337.
63. a. Roessner U, Wagner C, Kopka J, Trethewey RN, Willmitzer L. 2000. Simultaneous analysis of metabolites in potato tuber by gas chromatography-mass spectrometry. *Plant J* **23**: 131–142.
    b. Roessner U, Luedemann A, Brust D, *et al.* 2001. Metabolic profiling allows comprehensive phenotyping of genetically or environmentally modified plant systems. *Plant Cell* **13**: 11–29.
64. Sauter H, Lauer M, Fritsch H. 1991. Metabolic profiling of plants – a new diagnostic technique. *ACS Symp Ser* **443**: 288–299.
65. Schmidt K, Carlsen M, Nielsen J, Villadsen J. 1997. Modelling isotopomer distributions in biochemical networks using isotopomer mapping matrices. *Biotechnol Bioeng* **55**: 831–840.
66. Schuster S, Dandekar T, Fell DA. 1999. Detection of elementary flux modes in biochemical networks: a promising tool for pathway analysis and metabolic engineering. *TIBTECH* **17**: 53–60.
67. Schuster S, Fell DA, Dandekar T. 2000. A general definition of metabolic pathways useful for systematic organization and analysis of complex metabolic networks. *Nat Biotechnol* **18**: 326–332.
68. Shannon CE. 1997. The mathematical theory of communication (Reprinted). *M D Computing* **14**: 306–317.
69. Shetty HU, Holloway HW, Rapoport SI. 1995. Capillary gas chromatography combined with ion trap detection for quantitative profiling of polyols in cerebrospinal fluid and plasma. *Anal Biochem* **224**: 279–285.
70. Smedsgaard J, Frisvad JC. 1996. Using direct electrospray mass spectrometry in taxonomy and secondary metabolite profiling of crude fungal extracts. *J Microbiol Meth* **25**: 5–17.
71. Sobczak A, Skop B, Kula B. 1999. Simultaneous determination of serum retinol and alpha- and gamma-tocopherol levels in type II diabetic patients using high-performance liquid chromatography with fluorescence detection. *J Chromatogr* **730**: 265–271.
72. Stein SE. 1999. An integrated method for spectrum extraction and compound identification from gas chromatography/mass spectrometry data. *J Am Soc Mass Spectrom* **10**: 770–781.
73. Szyperski T. 1998. 13C-NMR, MS and metabolic flux balancing in biotechnology research. *Quat Rev Biophys* **31**: 41–106.
74. Tam YY, Normanly J. 1998. Determination of indole-3-pyruvic acid levels in *Arabidopsis thaliana* by gas chromatography selected ion monitoring mass spectrometry. *J Chromatogr A* **800**: 101–108.
75. Tanaka K, Hine DG, West-Dull A, Lynn TB. 1980. Gas-chromatographic method of analysis of urinary organic acids. I. Retention indices of 155 metabolically important compounds. *Clin Chem* **26**: 1839–1846.
76. Tanaka K, West-Dull A, Hine DG, Lynn TB, Lowe T. 1980. Gas-chromatographic method of analysis of urinary organic acids. II. Description of the procedure, and its application to diagnosis of patients with organic acidurias. *Clin Chem* **26**: 1847–1853.
77. Teusink B, Baganz F, Westerhoff HV, Oliver SG. Metabolic control analysis as a tool in the elucidation of the function of novel genes. In *Methods in Microbiology*. Vol. 26, Academic Press; 297–336.
78. Thiellement H, Bahrmann N, Damerval C, *et al.* 1999. Proteomics for genetic and physiological studies in plants. *Electrophoresis* **20**: 2013–2026.
79. Thomas S, Mooney PJF, Burrell MM, Fell DA. 1997. Metabolic control analysis of glycolysis in tuber tissue of potato (Solanum tuberosum): explanation for the low control coefficient of phosphofructokinase over respiratory flux. *Biochem J* **332**: 119–127.
80. Trethewey RN, Krotzky AJ, Willmitzer L. 1999. Metabolic profiling: a Rosetta stone for genomics? *Curr Opin Plant Bio* **2**: 83–85.
81. Tweeddale H, Notley-McRobb L, Ferenci T. 1998. Effect of slow growth on metabolism of *Escherichia coli*, as revealed

Copyright © 2001 John Wiley & Sons, Ltd. 

by global metabolite pool ('Metabolome') analysis. *J Bacteriol* **180**: 5109–5116.
82. Varner J, Ramkrishna D. 1999. Mathematical models of metabolic pathways. *Curr Opin Biotechnol* **10**: 146–150.
83. Velot C, Mixon MB, Teige M, Srere PA. 1997. Model of a quinary structure between Krebs TCA cycle enzymes: A model for the metabolon. *Biochemistry* **36**: 14271–14276.
84. Warne MA, Lenz EM, Osborn D, Weeks JM, Nicholson JK. 2000. An NMR-based metabonomic investigation of the toxic effects of 3-trifluoromethyl-aniline on the earthworm *Eisenia veneta*. *Biomarkers* **5**: 56–72.
85. WIT **http://wit.mcs.anl.gov/WIT2/**
86. Wolfender JL, Rodriguez S, Hostettmann K. 1998. Liquid chromatography coupled to mass spectrometry and nuclear magnetic resonance for the screening of plant constituents. *J Chromatog A* **794**: 299–316.
87. World Health Organization. 2000. Safety aspects of genetically modified foods of plant origins. Report of a joint FAO/WHO expert consultation on foods derived from biotechnology, held in Geneva, Switzerland, 29 May–2 June 2000. *World Health Organization* 1–35.

*The*
**Genomics**
*Website at Wiley*

**www.wiley.co.uk/genomics**

The **Genomics** website at Wiley is a **DYNAMIC** resource for the genomics community, offering **FREE** special feature articles and new information **EACH MONTH**.

Find out more about our new journals *Comparative and Functional Genomics,* and *Proteomics.*

Visit the **Library** for hot books in Genomics, Bioinformatics, Molecular Genetics and more.

Click on **Journals** for information on all our up-to-the minute journals, including: *Genesis, Bioessays, Journal of Mass Spectrometry, Gene Function and Disease, Human Mutation, Genes, Chromosomes and Cancer* and the *Journal of Gene Medicine.*

Let the **Genomics** website at Wiley be your **guide** to genomics-related web sites, manufacturers and suppliers, and a calendar of conferences.

WILEY

2380

Copyright © 2001 John Wiley & Sons, Ltd. 
